# GPR55 senses lactate to sustain motility in prostate cancer cells

**DOI:** 10.1007/s11010-025-05312-0

**Published:** 2025-05-28

**Authors:** Giovanna Sgrignani, Marta Iozzo, Lara Di Leonardo, Elisa Pardella, Erica Pranzini, Giulia Gangarossa, Giuseppina Comito, Luigi Ippolito, Elisa Giannoni, Paola Chiarugi

**Affiliations:** Department of Experimental and Clinical Biomedical Sciences, “Mario Serio”. University of Florence, Viale Morgagni 50, 50134 Florence, Italy

**Keywords:** Prostate cancer, Lactate, GPR55, Amoeboid motility

## Abstract

The enrichment of specific metabolites within the tumor microenvironment is emerging as a driver of tumor progression. Specifically, in prostate cancer (PCa), increased abundance of lactate is associated with primary-to-metastasis tumor spreading by supporting cancer cell invasiveness. Here, we highlight that the endocannabinoid receptor GPR55 is able to sense lactate and consequently trigger PCa cell amoeboid-like invasiveness, through the activation of the pro-migratory RhoA/MLC2 signaling pathway. These findings uncover a new role for GPR55 in sustaining lactate-driven PCa cell motility.

**Supplementary Information:**

The online version contains supplementary material available at 10.1007/s11010-025-05312-0.

## Introduction

Lactate is the main product of fermentative metabolism of cancer and cancer-associated cell populations (e.g., cancer-associated fibroblasts, CAFs), frequently accumulated in tumor microenvironment (TME) [1, 2].

Lactate transport is mediated by both protons (monocarboxylate transporters, *i.e.,* MCT1-4) and sodium-dependent co-transporters (SLC5 A8 and SLC5 A12). The transport direction depends on the lactate gradient, leading to preferential lactate import within the TME. The concentration of lactate has been found to range between 10 and 40 mM within solid tumors [3, 4], while levels of circulating lactate vary from 1 to 2 mM. High extracellular lactate levels have been associated to tumor patient worse prognosis, as it reprograms cancer cell metabolism and sustains cancer cell invasion, stemness and immune escape [5–9]. Recent findings have highlighted new unconventional roles of lactate as transcriptional driver mediating epigenetic modifications such as histone acetylation and lactylation [10–12]. In addition to its metabolic exploitation, lactate also contributes to cancer progression by signaling through the GPR81 “*lactormone*” sensor at lower concentration [13].

In prostate cancer (PCa), CAFs represent one of the major contributors to lactate enrichment in the TME, establishing a lactate-driven metabolic coupling with PCa cells that sustains tumor progression [2]. The transition toward an activated, lactate-producing CAF phenotype is influenced by cytokines released by tumor cells [14] and, more recently, by an involvement of endocannabinoid receptors (CBRs, i.e., CB1-2Rs) [15].

Furthermore, physiological engagement of CB1 receptors regulates several cell behaviors, including neuronal energy metabolism. Notably, astrocytic CB1R stimulation controls brain lactate levels via lactate-sensing GPR81 receptor activation [16]. Deregulated CBRs activation in tumor cells underlies a tumor-promoting effect on cancer cell proliferation, invasiveness and the activation of intracellular signaling cascades [17–19]. In particular, the non-canonical CBR GPR55 has been shown to trigger intracellular calcium mobilization, which plays a key role in tumor progression [20]. Furthermore, GPR55 activation sustains MLC2 signaling [19], typical of amoeboid invasiveness. GPR55 is a G-protein-coupled receptor (GPCR) [21] whose activation by anandamide, 2-arachidonoylglycerol (2-AG) or lysophosphatidylinositol (LPI) as the main ligands resulted in higher tumor cell aggressiveness [22–24].

Given the role of environmental lactate in PCa motility [10], this observation prompted us to investigate the relationship between lactate and the CBRs in PCa. Hence, we underlined a novel role for lactate as an activator of GPR55 in PCa cells, supporting amoeboid-like tumor cell migration.

## Materials and methods

### Cell lines

DU145 (RRID: CVCL_0105), PC3 (RRID: CVCL_0035) and HEK293 T were purchased from ATCC and cultured in DMEM (#ECB7501L, Euroclone) with 10% FBS (#ECS5000L; Euroclone), 2 mmol/L L-glutamine (#G7513-100ML, Merck Sigma) and 1% penicillin/streptomycin (#P0781-100 ML, Merck Sigma) at 37 °C and 5% CO_2_. Cells were routinely tested for Mycoplasma contamination.

### Cell treatments and reagents

2.5 mM and 20 mM lactic acid (#L6402, Sigma-Aldrich) in serum-free medium were used to treat PCa cells for 15 min or 48 h, as detailed in the Figure Legends. ML193 (#SML1340, Sigma-Aldrich) was used at a final concentration of 5 μM for 30 min or 48 h, as detailed in the Figure Legends. The Rho inhibitor (CT04, Società Italiana Chimici Divisione Scientifica S.R.L.) and the broad-spectrum matrix metalloproteinases (MMPs) inhibitor, Marimastast (HY-12169, D.B.A s.r.l.), were used at 1 µg/ml and 10 µM final concentrations, respectively. LPI (#440,153, Sigma-Aldrich) was used at 10 µM final concentration. Cells were pre-treated with CT04 or Marimastat for 6 h or 24 h prior to the invasion assay, respectively.

### RNA extraction and real-time PCR

RNA extraction was performed by using the RNeasy Kit (#74,104; Qiagen). The iScript cDNA Synthesis Kit (#1,708,891; Bio-Rad) was utilized for cDNA synthesis. Real-Time PCR was carried out with the CFX96 Touch Real-Time PCR Detection System (Bio-Rad), using TaqMan assays (#4,440,040, Applied Biosystem). The following probes were used: CNR1 (#Hs01038522_s1), CNR2 (#Hs05019229_s1), GPR55 (#Hs00271662_s1), TRPV1 (#Hs00218912_m1) and GPR81 (#Hs02597779_s1). Data were normalized on HPRT1 (#Hs02800695_m1).

### Western blot analysis

Cells were lysed with the Laemmli buffer (#1,610,747, Bio-Rad) and protein content was quantified with the BCA Kit (#1,003,579,336, Sigma-Aldrich). 20–25 μg of samples were loaded on 4–20% acrylamide precast SDS-PAGE gels (#4,568,096, Bio-Rad) following the previously described procedures [10]. The following primary antibodies were used: rabbit anti-pMLC2 (1:1000; #3671S; Cell Signaling Technology), anti-MLC2 (1:1000; #3672S Cell Signaling Technology), anti-RhoA (1:1000; #sc-418, Santa Cruz Biotec) and mouse anti-HSP90 (1:1000; #sc-11818, Santa Cruz Biotec).

### Transfection

For GPR55 overexpression, HEK293 T cells were transfected with 1 µg cDNA/well using X-tremeGENE Transfection Reagent (#XTGHP-RO, Roche), according to the manufacturer instructions. The pcDNA3 empty vector or that coding for human hemagglutinin (HA)-GPR55 wild-type were used. The HA-GPR55 construct was a gift from Dr. Stefania Mariggiò, CNR, Naples (Italy).

### Calcium imaging

DU145 or HEK293 T cells were seeded on Nunc Lab-Tek II chambered Coverglass (#154526PK, Thermo Fisher Scientific) and, respectively, 48 h after 20 mM lactate treatment or 24 h after transfection, were synchronized for 4 h in serum-free medium. Then, cells were stained by using 2.5uM Fluo-4 AM dye (#F14201, Thermo Fisher Scientific) for 20 min at 37 °C. After a washing step, the indicated stimuli (2.5 mM lactate or 10 µM LPI) were added and fluorescence was recorded for 15 min using TCS SP8 microscope (Leica Microsystems) with LAS-AF image acquisition software. Ionomycin (5 µM) was used as a positive control for calcium rise.

### Immunoprecipitation (Rhotekin assay)

Cells were washed with phosphate buffered saline and then lysed in RIPA buffer (supplemented with 0.1% SDS) on ice. The cell lysates were centrifuged at 12,000 rpm and 4 °C for 20 min, and then incubated with 25 μg GST–Rhotekin beads (#14–383, Merck Life Science) at 4 °C for 45 min. Western blot analysis was performed to evaluate the expression of Rhotekin-bound Rho proteins (GTP-bound Rho), using the Rho A monoclonal antibody. The amount of Rhotekin-bound Rho was normalized to the total Rho present in the cell lysate.

### Transwell migration and invasion assay

Boyden chambers with 8 μm pore size filters (Costar™, Corning, #CC3422), coated or not with Matrigel, were used for the invasion and migration assays, respectively, as previously described [10]. At the end point, migrating and invading cells were fixed and then stained with Diff-Quick solution (BD Biosciences). Photos of five randomly chosen fields (10 × magnification) in bright field were taken for subsequent quantification of migrated/invaded cells by ImageJ software.

### Statistical analysis

Graph Pad software was used for statistical analysis. Data are reported as mean ± SEM from at least three independent experiments. Unpaired Student t-test (two-tailed), ordinary one-way ANOVA followed by Tukey’s correction, or two-way ANOVA followed by Sidak’s correction were performed for statistical comparison. Statistical significance (p-value): *, < 0.05; **, < 0.01; ***, < 0.001; ****, < 0.0001.

## Results

### Lactate drives the activation of GPR55 in PCa cells to promote amoeboid cell motility

**Fig. 1:**
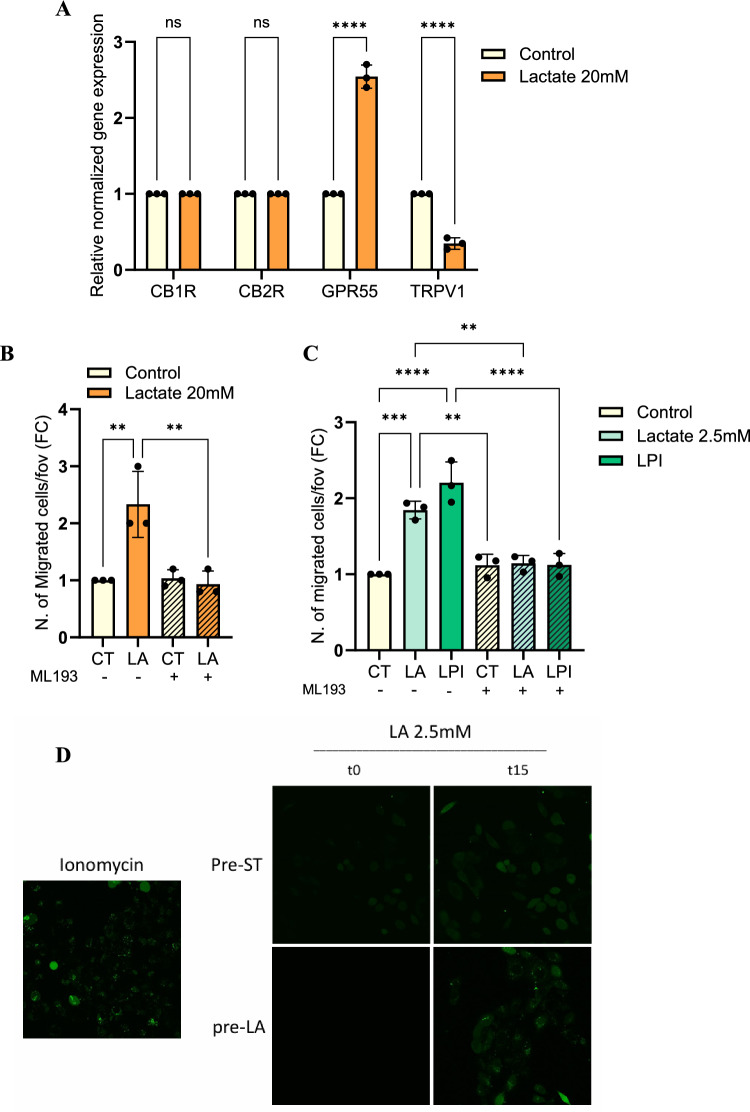
Lactate-sensing by GPR55 supports the migration ability of DU145 cells. **A** RT-PCR analysis for the CB1R, CB2R, GPR55 and TRPV1 expression in DU145 cells treated with 20 mM lactate for 48 h. Data are reported as Fold Change (FC) with respect to control. **B**–**C** 3 × 10.^4^ DU145 cells treated with 20 or 2.5 mM lactate and 10 µM LPI (C) for 48 h ± ML193 (5 µM) were allowed to migrate through transwell chambers for 16 h. **D** Representative confocal images showing the levels of calcium flux assessed in DU145 cells after a 48 h-pretreatment with lactate 20 mM and, upon refresh with serum-free medium, the subsequent stimulation with lactate (2.5 mM); ionomycin (5 µM) was reported as positive control (magnification 40 ×; time = 0 and 15 min post-stimulation). All data are represented as the mean ± SEM of *n* = *3* experiments. Two-way ANOVA following Sidak’s multiple comparisons (A); One-way ANOVA following Tukey’s corrected (B-C). * p < 0.05; ** p < 0.01; ***p < 0.001; **** p < 0.0001

To assess whether a lactate-rich environment may alter ECS players in PCa, we exposed DU145 cells to 20 mM lactate for 48 h and then we evaluated the expression of the main CBRs (CB1-2Rs, TRPV1, GPR55). Real-time PCR analysis revealed that GPR55 and TRPV1 are positively and negatively regulated by lactate, respectively, while the expression of CB1R and CB2R result unaffected (Fig. 1A).

Therefore, we sought to dissect whether a lactate-GPR55 axis is involved in PCa cell motility. Firstly, we evaluated the migratory ability of DU145 cells exposed to high and low lactate concentrations (20 and 2.5 mM), reported to mimic the concentration found in TME and able to activate GPCR [25, 26], respectively, and in the presence of ML193 inhibitor to pharmacologically interfere with GPR55. Importantly, we observed increased cell migration of DU145 cells treated with both lactate concentrations, which is prevented by the incubation with ML193, suggesting a potential lactate-induced GPR55 activation that enhances cancer cell motility (Fig. 1B–C). To further strengthen the role of lactate in activating GPR55-mediated response, we compared the effects of lactate exposure to those induced by LPI (10 µM), a known endogenous GPR55 ligand. Interestingly, we observed that LPI stimulation recapitulates the lactate-induced increase in DU145 cell migration, and that this effect is reversed by ML193 (Fig. 1C). These findings reinforce the notion that lactate can functionally engage GPR55, resulting in responses similar to those established by its known agonists. To note, we excluded the involvement of GPR81 receptor as it is not expressed in our PCa model (Figure S1A).

To assess whether lactate directly activates GPR55 signaling and to identify signaling pathways induced upon lactate-dependent GPR55 stimulation, we assessed the ability of lactate to elicit calcium mobilization, a known GPR55 downstream signaling [27]. DU145 cells (previously exposed to lactate 20 mM for 48 h to increase GPR55 expression) were short-pulsed (15 min) with lactate 2.5 mM to elicit GPR55 activation. Using live-cell confocal imaging, we observed that short-term lactate stimulation triggers a rapid increase in intracellular calcium levels (Fig. 1D). To further confirm the involvement of GPR55 in this response, we performed a heterologous transient overexpression of GPR55 in HEK293 T cells (which normally express low levels of GPR55) (Figure S1B). Calcium flux analysis showed that GPR55-overexpressing (OE) cells similarly respond to lactate and LPI stimulation, while mock-transfected cells display minimal response (Figure S1C), reinforcing the specificity of the observed effects from GPR55 activation.

**Fig. 2:**
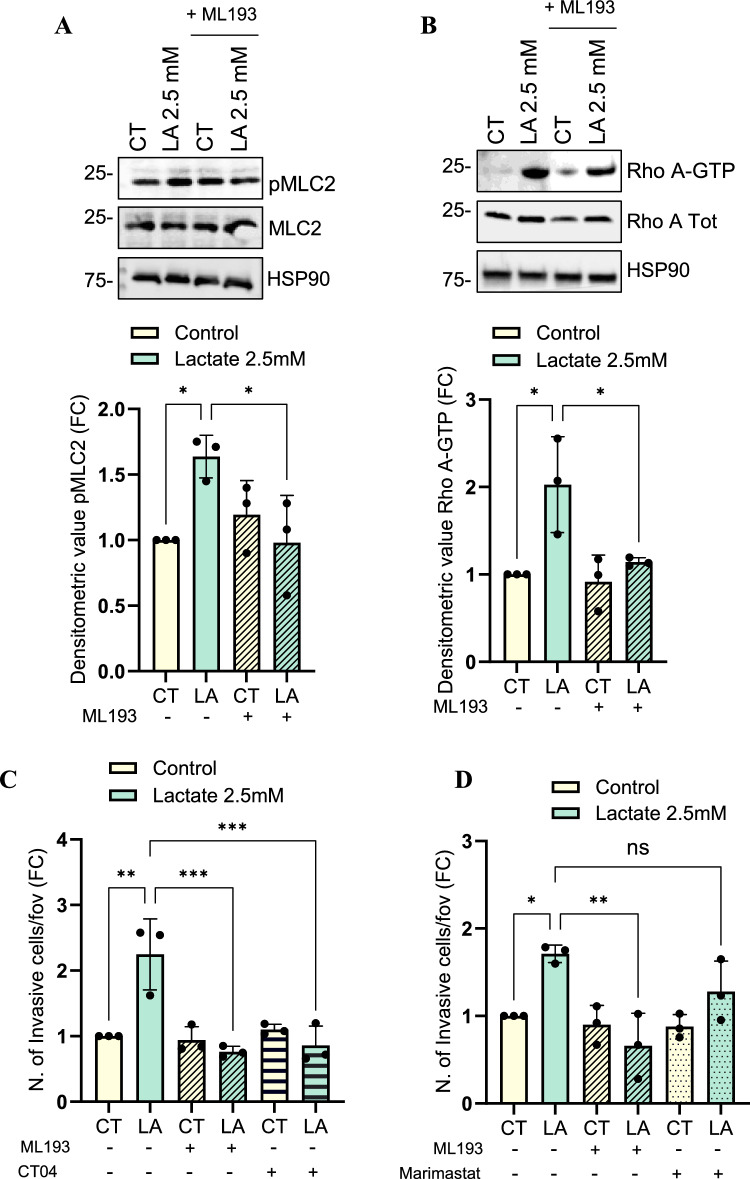
Lactate-mediated activation of GPR55 promotes the amoeboid motility in DU145 cells via RhoA/MLC2 signaling. **A** Total protein lysates from DU145 cells treated with 2.5 mM lactate for 15 min ± ML193 (5 µM) were subjected to WB analysis for evaluating pMLC2 and total MLC2 protein levels. HSP90 was used as housekeeping protein. **B** WB analysis of GTP-bound RhoA was performed as described in Method section. GTP-Rho levels were normalized on total Rho and HSP90 proteins. **C** 5 × 10^4^ DU145 cells, treated with 2.5 mM lactate for 48 h and administered with CT04 (1 μg/mL) or ML193 (5 μM), **D** or with Marimastat (10 μM) or ML193 (5 μM), as described in the Method section, were allowed to invade through matrigel-coated transwell chambers for 16 h. All data are represented as the mean ± SEM of *n* = *3* experiments. One-way ANOVA following Tukey’s corrected (A-E). * p < 0.05; ** p < 0.01; ***p < 0.001

We also observed that short-term lactate stimulation increases MLC2 phosphorylation (pMLC2) in PCa cells, which is counteracted by GPR55 inhibition with ML193, suggesting that lactate could regulate MLC2 activation via GPR55 (Fig. 2A). This observation, also corroborated in the PC3 cell line (Figure S1D), strengthens the idea that lactate may act not only as a metabolite but also as a GPR55 *‘agonist’* to activate MLC2 signaling.

MLC2 is the downstream molecule of Rho-ROCK signaling that regulates the amoeboid migration [28–30]. To corroborate that lactate is involved in triggering the Rho/MLC2 signaling downstream of GPR55 activation, we found that lactate sustains RhoA activation in DU145 cells by increasing the GTP-bound active form of RhoA, which is essential to coordinate cytoskeleton rearrangement during amoeboid cell motility. Notably, ML193-induced GPR55 inhibition reduces the lactate-mediated RhoA switch into its active form (Fig. 2B).

Amoeboid cells do not likely need the proteolytic degradation of extracellular matrix, a well-established feature of mesenchymal invasive cells [29, 31–33]. To exclude the mesenchymal invasion, we assayed the invasive ability of lactate-exposed PCa cells by using the Rho and matrix metalloproteinases (MMPs) inhibitors, CT04 and Marimastat, respectively. RhoA targeting strongly reduces lactate-stimulated PCa cell invasiveness (Fig. 2C, S1E), while MMPs blockade does not significantly decrease PCa cell invasive ability (Fig. 2D), suggesting that lactate-sensing PCa cells are likely to adopt rounded-amoeboid movement. Of note, we observed that GPR55 inhibition exerts an anti-invasive effect on lactate-treated cells (Fig. 2C, S1E), highlighting that the activation of lactate-GPR55 circuit correlates with the engagement of an amoeboid phenotype.

Overall, these findings identify lactate as a signaling molecule sensed by GPR55, which induces PCa cells to acquire an amoeboid motility via RhoA/MLC2 activation.

## Discussion

Lactate likely exerts pleiotropic effects on cell motility, with its impact varying according to the concentration of intra- and extracellular lactate. Indeed, a lactate-rich environment (10–20 mM), commonly observed at the primary tumor due to hypoxia and to the high glycolytic activity of cancer and surrounding accessory cells, has been reported to trigger malignant and non-malignant cell invasion/migration by altering genetic and epigenetic cellular profile [10, 12, 34]. Particularly, transcriptional program for a mesenchymal MMP-dependent motility (i.e., EMT) is triggered in myocardial cells through Snail1 lactylation following myocardial infarction [35] as well as in thyroid and renal carcinomas through LDHA- or Sirtuin 1-dependent mechanisms [36–38]. In keeping, PCa cells acquire a metastatic phenotype due to upload of high level of stromal lactate [10]. However, cancer cells are exposed to different concentrations of lactate along the metastatic cascade which can elicit cancer aggressiveness through differential mechanisms. Specifically, circulating cancer cells are exposed to lower levels of lactate (1.5–5 mM in the bloodstream), which have been reported to similarly elicit pro-tumoral behaviors, including enhanced motility, while acting through different mechanisms by binding to membrane receptors, like GPR81 [13, 25, 39–41]. However, those studies did not deeply investigate which type of cell movement is specifically regulated by a low amount of extracellular lactate.

Herein, we report a novel role for lactate behaving as a driver of the MMP-independent amoeboid motility. Specifically, we observed that lactate at low concentration may act through a membrane receptor already known to interact with endocannabinoids, GPR55, to enhance cellular motility via RhoA/MLC2 activation in PCa cells. The here suggested dual role of high-low extracellular lactate in inducing different cell migratory strategies provides cancer cells with a particular plasticity in adopting mesenchymal or amoeboid migration modes [42] in response to the dynamic metabolic (i.e., lactate, hypoxia) environment they face with. Indeed, high levels of lactate sensed by cancer cells at the primary tumor [10], may facilitate a mesenchymal invasion of surrounding tissues to reach the bloodstream and start the metastatic route [29]. Differently shifting to an amoeboid-like phenotype, previously associated with trans-endothelial migration [31] may enable tumor cells to fast squeeze through the extracellular matrix of distant organs [32, 33], generally characterized by lower levels of lactate than tumor tissues.

This study highlights a novel role of lactate in the complex mechanisms underlying tumor progression and metastasis and identifies GPR55 as an additional lactate membrane receptor involved in cancer cell motility. Pharmacological inhibition of GPR55 has provided promising results across various cancer models. In colorectal cancer, the selective antagonist CID16020046 impairs cell adhesion and migration in vitro and reduces liver metastases in vivo, supporting a role for GPR55 in tumor cell dissemination [43]. Similarly, in pancreatic cancer, the GPR55 inhibitor (R,R’)−4’-methoxy-1-naphthylfenoterol (MNF) suppresses tumor proliferation and enhances sensitivity to chemotherapeutics such as doxorubicin and gemcitabine [44, 45]. These findings highlight GPR55 as a potential therapeutic target to curb tumor spread and metastasis across different cancer types. However, further investigations are required to elucidate the lactate’s role in the final stages of the metastatic cascade, as well as to explore the potential competitive interactions between endocannabinoids and lactate in modulating GPR55 activation.

## Supplementary Information

Below is the link to the electronic supplementary material.

Supplementary file1 (DOCX 176 KB)

## Data Availability

No datasets were generated or analysed during the current study.
