# Supplementary material for: GPR55 senses lactate to sustain motility in prostate cancer cells
Source: Mol Cell Biochem. 2025 May 28;480(9):5197–204. doi: 10.1007/s11010-025-05312-0 (PMC12476429; doi:10.1007/s11010-025-05312-0)
Supplement: Supplementary file 1 — Supplementary file1 (DOCX 176 KB) [file 11010_2025_5312_MOESM1_ESM.docx]

**GPR55 senses lactate to sustain motility in prostate cancer cells**

Giovanna Sgrignani^1^*, Marta Iozzo^1^*, Lara Di Leonardo^1^, Elisa Pardella^1^, Erica Pranzini^1^, Giulia Gangarossa^1^, Giuseppina Comito^1^, Luigi Ippolito^1^**, Elisa Giannoni^@1^, Paola Chiarugi^@1^

*^1^ Department of Experimental and Clinical Biomedical Sciences, “Mario Serio”. University of Florence, Viale Morgagni 50, 50134, Florence, Italy*

******Equal contribution as first authors*

**^@^***Equal contribution as last authors*

***Corresponding author*: L. Ippolito, luigi.ippolito@unifi.it

**Supplementary Material**


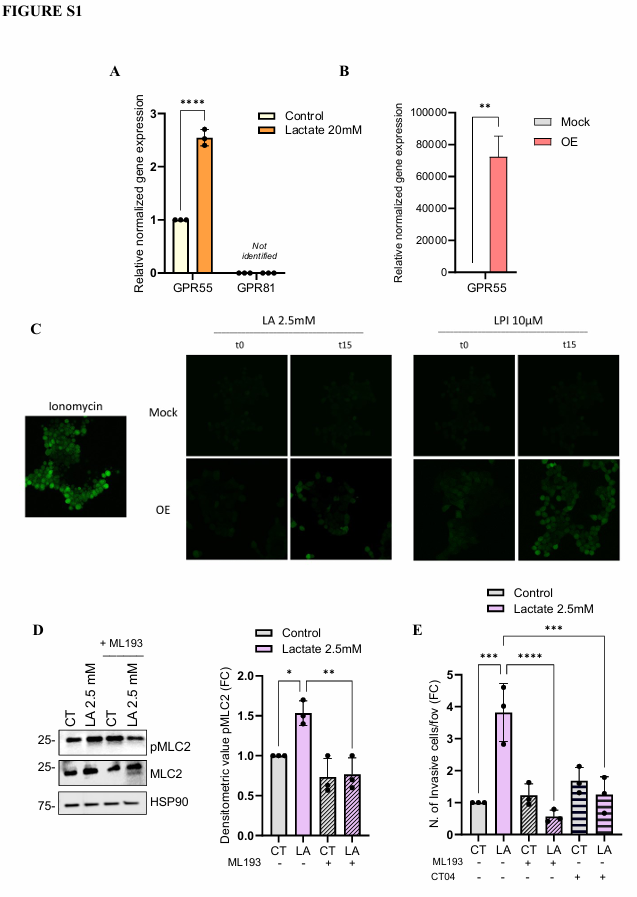


**Supplementary Fig. 1**

Lactate promotes GPR55 activation and RhoA-dependent invasiveness in PC3 cells. **A)** RT-PCR analysis for the GPR55 and GPR81 in DU145 cells treated as figure 1A. **B)** RT-PCR analysis for GPR55 in HEK293T cells transfected with GPR55-expressing plasmid versus mock-transfected controls. **C)** Representative confocal images showing the levels of intracellular calcium flux assessed after stimulation with lactate (2.5 mM) or LPI (10 µM) in GPR55-OE and mock-transfected HEK293T cells. Ionomycin (5 µM) was reported as positive control (magnification 40×; time = 0 and 15 min post-stimulation). **D)** Total protein lysates from PC3 cells, treated with 2.5 mM lactate for 15 min ± ML193 (5 µM) were subjected to WB analysis of pMLC2 and total MLC2. HSP90 was used as housekeeping protein. **E)** 5x10^4^ PC3 cells, treated with 2.5 mM lactate for 48 hours and administered with CT04 (1 µg/mL) or ML193 (5 µM) were allowed to invade through matrigel-coated transwell chambers for 16h. All data are represented as the mean ± SEM of *n=3* experiments. Two-way ANOVA following Sidak’s multiple comparisons (A); One-way ANOVA following Tukey’s corrected (B, D, E). * p<0.05; ** p<0.01; ***p<0.001; **** p<0.0001
